# Identification of Common Biological Pathways and Drug Targets Across Multiple Respiratory Viruses Based on Human Host Gene Expression Analysis

**DOI:** 10.1371/journal.pone.0033174

**Published:** 2012-03-14

**Authors:** Steven B. Smith, William Dampier, Aydin Tozeren, James R. Brown, Michal Magid-Slav

**Affiliations:** 1 Department of Bioengineering, University of Pennsylvania, Philadelphia, Pennsylvania, United States of America; 2 Computational Biology, Quantitative Sciences, GlaxoSmithKline, King of Prussia, Pennsylvania, United States of America; 3 Center for Integrated Bioinformatics, Drexel University, Philadelphia, Pennsylvania, United States of America; 4 Computational Biology, Quantitative Sciences, GlaxoSmithKline, Collegeville, Pennsylvania, United States of America; Faculty of Biochemistry Biophysics and Biotechnology, Jagiellonian University, Poland

## Abstract

**Background:**

Pandemic and seasonal respiratory viruses are a major global health concern. Given the genetic diversity of respiratory viruses and the emergence of drug resistant strains, the targeted disruption of human host-virus interactions is a potential therapeutic strategy for treating multi-viral infections. The availability of large-scale genomic datasets focused on host-pathogen interactions can be used to discover novel drug targets as well as potential opportunities for drug repositioning.

**Methods/Results:**

In this study, we performed a large-scale analysis of microarray datasets involving host response to infections by influenza A virus, respiratory syncytial virus, rhinovirus, SARS-coronavirus, metapneumonia virus, coxsackievirus and cytomegalovirus. Common genes and pathways were found through a rigorous, iterative analysis pipeline where relevant host mRNA expression datasets were identified, analyzed for quality and gene differential expression, then mapped to pathways for enrichment analysis. Possible repurposed drugs targets were found through database and literature searches. A total of 67 common biological pathways were identified among the seven different respiratory viruses analyzed, representing fifteen laboratories, nine different cell types, and seven different array platforms. A large overlap in the general immune response was observed among the top twenty of these 67 pathways, adding validation to our analysis strategy. Of the top five pathways, we found 53 differentially expressed genes affected by at least five of the seven viruses. We suggest five new therapeutic indications for existing small molecules or biological agents targeting proteins encoded by the genes F3, IL1B, TNF, CASP1 and MMP9. Pathway enrichment analysis also identified a potential novel host response, the Parkin-Ubiquitin Proteasomal System (Parkin-UPS) pathway, which is known to be involved in the progression of neurodegenerative Parkinson's disease.

**Conclusions:**

Our study suggests that multiple and diverse respiratory viruses invoke several common host response pathways. Further analysis of these pathways suggests potential opportunities for therapeutic intervention.

## Introduction

Respiratory viruses account for seasonal colds, bronchiolitis, acute otitis, sinusitis, croup, community-acquired pneumonia, and exacerbation of both chronic obstructive pulmonary disease and asthma [1]. The prevalence of pandemic *Orthomyxoviridae* Influenza A Virus (FLU) from April 2009 to 2010 was estimated to be approximately 60 million cases, 270,000 hospitalizations, and 12,000 deaths [2]. *Paramyxoviridae* Respiratory Syncytial Virus (RSV) infection results in nearly two million children requiring medical care with about 57,000 children younger than five years hospitalized annually [3]. In one survey, RSV was the most prevalent pathogen in children under five years with an acute respiratory infection, followed by *Adenoviridae* adenovirus (ADENO), and *Picornaviridae* human rhinovirus (HRV) [4].

While initially effective, pathogen gene targeted treatments exert evolutionary selection on the infectious species often leading to the emergence of drug resistant strains. As a result, there are increasing clinical reports of resistance against many drugs that directly act on viral proteins or their DNA [5], [6]. In particular, resistance to different classes of antiviral drugs is becoming more clinically prevalent in respiratory virus infections as seen with RSV and FLU treated with the antiviral drugs palivizumab [7], and oseltamivir [8], respectively.

Pathogens elucidate two broad types of biochemical responses in the host. First is the activation of the host immune system. While the immune response is critical in combating pathogen infections, its over-activation often exacerbates tissue damage initiated by viral invasion [9], [10]. The second response is the up-regulation of host genes, such as protein biosynthetic pathways, that are crucial for sustaining pathogen invasion, replication and evasion [11]. Interestingly, genetically distinct respiratory viruses often modulate common host proteins and biological pathways during infection [1]. For example, many respiratory viruses trigger similar general airway inflammatory responses such as the expression of cytokines interleukin-6 (HUGO gene name IL6), interleukin-8 (IL8) and interleukin-11 (IL11), and granulocyte macrophage-colony stimulating factor (CSF2). These inflammatory responses in turn initiate IgA production, B cell differentiation and T cell stimulation [12]–[16]. As a consequence, diagnosis for specific viral infections is difficult since diverse respiratory viruses cause similar, often indistinguishable patient symptoms [1]. However, because distinct respiratory viruses converge on similar immune responses, opportunities also exist for targeting host proteins and pathways which will potentially affect multiple viral pathogens [17]. Moreover, human targets might be less susceptible to the evolution of drug resistance due to constraints on the virus to find alternative host pathways for its proliferation.

Individuals may experience a co-infection or sequential infections of multiple viruses or bacteria which can complicate both disease diagnosis and drug prescription decisions. Furthermore, patients infected by multiple pathogens may have further complications due to drug-drug interactions, cumulative drug toxicities and immune system suppression, as observed during HIV and *Mycobacterium tuberculosis* co-infections [18], [19]. Indeed, a study in children under five years showed pervasive clinical occurrences of co-infections involving combinations of RSV, HRV, *Paramyxoviridae* Parainfluenza Virus, FLU, *Coronaviridae* SARS-Coronavirus (CORON), *Paramyxoviridae* Metapneumonia virus (MPNEU), *Parvoviridae* Human Bocavirus and ADENO [4]. Therefore, in addition to minimizing drug resistance, there is a need for new therapeutic approaches to safely and effectively treat co-infections by multiple viral and/or bacterial pathogens, particularly where strain-specific diagnostics or treatments are unavailable.

The development of new antiviral therapeutics requires a greater understanding of the global host response when challenged by different types of viruses. Such knowledge may lead to the identification of novel human genome targets that are shared across multiple viral infections as well as opportunities for re-positioning existing drugs for the treatment of infectious diseases [20]. Several recent studies have generated multiple mRNA microarray gene expression datasets derived from experiments involving the infection of human cell-lines or animal models with one or more of the major respiratory viruses [21]–[23]. Through a systematic analysis of these respiratory virus-human host gene expression datasets, we determined common sets of genes and pathways involved in host responses to viral infections. Among the most significant pathways, we identified several potential new opportunities for repurposing existing drugs for the treatment of respiratory viral infections.

## Results

### Selection of mRNA Microarray Datasets

**Figure 1:**
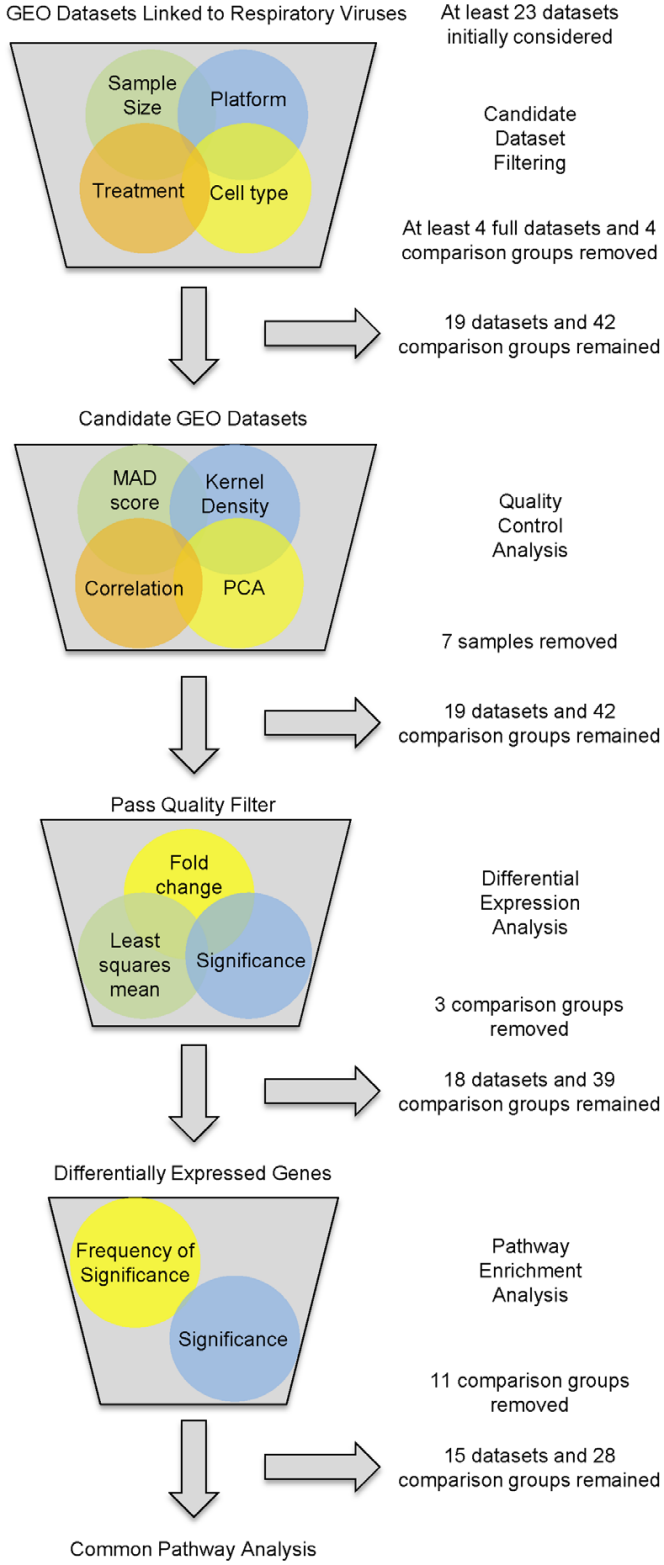
Outline of iterative filtering process. Analysis pipeline to select and quality control GEO datasets linked to respiratory virus mRNA expression. Specific inclusion criteria are described in the Materials and Methods.

We performed a large-scale analysis of published mRNA microarray datasets from studies involving a wide range of respiratory viruses in human host infection models. We focused on human mRNA array datasets in order to avoid complications inherent in cross-species comparisons. In order to ensure consistency in experimental conditions and reduce biases due to noisy or poor quality datasets, we instituted an iterative process of database querying, data filtering, and common pathway analysis across all published human mRNA datasets for twelve relevant respiratory viruses. These viruses initially included the double stranded DNA viruses *Herpesviridae* Human cytomegalovirus (CMV) and ADENO; the positive sense single stranded RNA viruses CORON, *Picornaviridae* Coxsackievirus (COX), HRV, *Picornaviridae* Echovirus (ECHO), and *Picornaviridae* Enterovirus (ENTERO); and the negative sense single stranded RNA viruses FLU, MPENU, RSV, *Bunyaviridae* Hantavirus (HANT) and Sin nombre virus (SNV). This list was later narrowed to include only the subset listed in Table 1 based on filtering processes outlined in the Materials and Methods and shown in Figure 1.

**Table 1:** Profiles of GEO datasets passing all criteria filters.

Virus Type	GSE Accession	Reference	Array Platform^1^	Cell type^2^	Time (hours)^2^	Sample Size^3^
CMV	24238	[104]	HG U95 v 2.0	Monocytes	24	3/3
	14816	[105]	HG U133 A	moDC	48	3/3
	11408	[106]	HG U95 v 2.0	Monocytes	4	6/6
	14490	N/A	Agilent G4112F	moDC	6	8/6
					48	6/6
CORON	1739	[107]	HG Focus	PBMC	N.S.^***^	10/4
	17400	[108]	HG U133 Plus 2	BEC (2B4)	12	3/3
					24	3/3
					48	3/3
COX	697	N/A	HG U95 v 2.0	HeLa	0.5	6/3
					3	5/3
					9	6/3
FLU	19580	[109]	Illumina Human Ref 8, version 3	BEC	24^4^	3/3
					24^5^	3/3
					6^4^	3/3
					6^5^	3/3
	17156	[110]	HG U133 A 2.0	Whole blood	80	8/8
	18816	[111]	HG 1.0 ST	Diff. macrophage	6	3/3
HRV	11348	[112]	HG U133 Plus 2	Nasal	48	31/31
	13396	[113]	HG U133 Plus 2	BEC	16	6/6
MPNEU	8961	[114]	HG U133 Plus 2	ABEC(A549)	6	3/3
					12	3/3
					24	3/3
					48	3/3
					72	3/3
RSV	17156	[110]	HG U133 A 2.0	Whole blood	141	9/9
	6802	[115]	HG U133 A 2.0	BEC (BEAS-2B)	4	3/3
	3397	[116]	HG U133 Plus 2	BEC (BEAS-2B)	4	4/4

1 Microarray manufacturer is Affymetrix unless otherwise noted.

2 N.S. = Not specified; moDC = Monocyte-derived dendritic cells;PBMC = Peripheral blood mononuclear cells; BEC = bronchial epithelial cells; ABEC = Alveolar BEC.

3 Before individual sample removal during quality control filtering. Sample Size refers to the number of GSM samples per treatment group versus control group.

4 Influenza A H3N2.

5 Influenza A H11N9.

A total of seven different respiratory viruses were analyzed, represented by fifteen unique Gene Expression Omnibus (GEO) datasets (indicated by GEO Series or GSE accession numbers), nine different human cell types, and seven different array platforms for a total of 28 unique comparisons. Note that one dataset (GSE17156) contained two different viruses (FLU and RSV) that were analyzed.

### Candidate Dataset Filtering and Quality Control

After querying the GEO database and prescreening for obvious non-candidate datasets such as those not associated with human array platforms, there were at least 23 datasets associated with at least one of the twelve respiratory viruses. However, after considering all conditions for GEO dataset candidacy, at least four of these datasets were excluded. In one case, an ADENO dataset (GSE1291 [PMID unpublished]) had less than three samples per treatment group, as did a COX (GSE712 [PMID unpublished]) and a CMV (GSE19345 [24]) dataset. As another example, a CMV dataset (GSE675 [25]) lacked a healthy/control treatment group. Additionally, at least four datasets had some comparison groups that did not fit the filters for inclusion. For instance, an HRV (GSE13396) dataset's original study design was to observe differences in HRV infectivity between asthmatic and non-asthmatic patients. The asthmatic comparison group data were eliminated from the analysis because of potential difficulties in distinguishing between host inflammatory responses due to viral infections from those associated with chronic asthma. Similarly, a combined FLU, HRV and RSV dataset (GSE17156) contained two main patient groups. One group was classified as developing symptoms after exposure to a single virus under study, while the other group did not develop any symptoms after exposure. Only the group that developed symptoms for each of the three viruses was considered for further analysis and the asymptomatic group was omitted. In total, 19 GEO datasets, representing 42 unique comparisons (different time points and/or virus strains) were analyzed for quality because they met the four requirements for dataset candidacy.

No single dataset exhibited overall poor Quality Control (QC), and therefore, all 19 datasets representing 42 comparison groups were analyzed for differential expression. However, QC analysis across all candidate datasets revealed two outliers in GSE17156 (samples GSM429252 and GSM429279), two in GSE11348 (samples GSM286647 and GSM286733), and one outlier each in dataset GSE24132 [26] (sample GSM594166), GSE1739 (sample GSM30367), and GSE19580 (sample GSM487986) for a total of seven samples removed from five different datasets.

**Figure 2:**
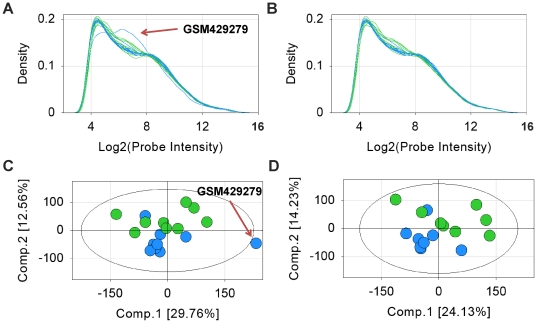
Example of quality analysis for subset of GSE17156: RSV treatment and control groups. Kernel density plot a) before and b) after removal of samples GSM429279 and GSM429252: blue lines indicate baseline samples, green lines indicate RSV peak symptom samples (∼141 hours); PCA plot c) before and d) after removal of samples GSM429279 and GSM429252 :blue circles indicate control (baseline) samples, green circles indicate RSV peak symptom samples (∼141 hours). Ellipse represents Hotelling T2 alpha threshold of 0.05. Eigenvalues for panel c components 1 and 2 are, respectively, 119335.7 and 50356.11. Eigenvalues for panel d components 1 and 2 are, respectively, 86014.46 and 50705.63.

An illustration of the kernel density and Principle Component Analysis (PCA) plots generated during the QC analysis is shown in Figure 2 for GSE17156's RSV treatment (median of 141 hours post infection) and RSV control (baseline) groups. Additional QC analysis results including Median of Absolute Deviation (MAD) score plots and pair-wise correlation maps are shown in Figure S1. Initially, all samples except GSM429279 showed acceptable kernel density (Figure 2a), PCA (Figure 2c), MAD score (Figure S1a) and pair-wise correlation (Figure S1c) plots. The sample GSM429279 was removed because: a) it did not conform to the kernel density of the other samples; b) it fell outside of the Hotelling T2 alpha threshold of 0.05 (represented by the superimposed elliptical on the PCA plot), and; c) it was an outlier in both the MAD score and pair-wise correlation plots. A second QC round was performed, which resulted in a further non-conforming sample, GSM429252, being discarded. Subsequent QC analysis generated acceptable results in kernel density (Figure 2b), PCA (Figure 2d), MAD score (Figure S1b), and pair-wise correlation (Figure S1d), thus this dataset passed our criteria for inclusion in the analysis.

### Differential Expression Analysis

All datasets exhibiting acceptable quality were analyzed for probe differential expression. An example volcano plot is shown in Figure S2 for RSV treatment group at peak symptoms versus control group (data originating from GSE17156). Cutoff levels of 1.5-fold increase or decrease in probe expression levels, respectively, and p-values <0.05 were used throughout (represented by red lines in Figure S2). All comparison groups had at least some differentially expressed probes, although the number varied greatly indicating potential falsely discovered probes (for example, a comparison group within GSE18816 had 111 differentially expressed probes while a comparison group within GSE11408 had 2533 differentially expressed probes). However, the conservative pathway enrichment approach we employed tends to attenuate falsely discovered genes.

There were three comparison groups that did not meet the Least Square Mean (LSM) threshold requirement and were excluded from the differentially expressed probe list: two of the only ENTERO comparison groups were from the GSE15323 [unpublished] dataset, and the third comparison group was an RSV treatment from GSE3397. After LSM filtering, 18 datasets, or a total of 39 comparison groups remained.

### Pathway Enrichment Analysis

For each comparison group, the differentially expressed probes were mapped to their corresponding genes, and then a p-value was assigned for each pathway map using the software GeneGo (accessed June 2011). Next, the comparison group's significant pathway lists were combined to find the union of all significant pathways (that is, the combined pathway list where all treatment groups have at least one significant pathway). A total of 459 out of the approximately 650 pathway maps available in MetaBase were determined to be significant. Comparison groups having <5% significant pathways of the total significant pathways (that is, comparison groups containing less than 23 significant pathways) lead to the exclusion of eleven comparison groups from the union list. Excluded groups were: HRV at 8 hours (eliminating one comparison group from GSE11348), HRV at 72 hours (eliminating one comparison group from GSE17156), both strains of FLU at 1 hour and 3 hours each and another strain at 6 hours (eliminating three comparison groups from GSE18816), RSV at 24 hours (eliminating all comparison groups from GSE24132), CMV at 24 and 72 hours (eliminating all comparison groups from GSE24434 [27]), and FLU at 8 hours (eliminating all comparison groups from GSE24533 [28]). At the end of the final step in our filtering process, a total of 15 datasets, or 28 comparison groups remained (Tables 1, S1 and S2).

### Common Pathways to Respiratory Viral Infection

**Figure 3:**
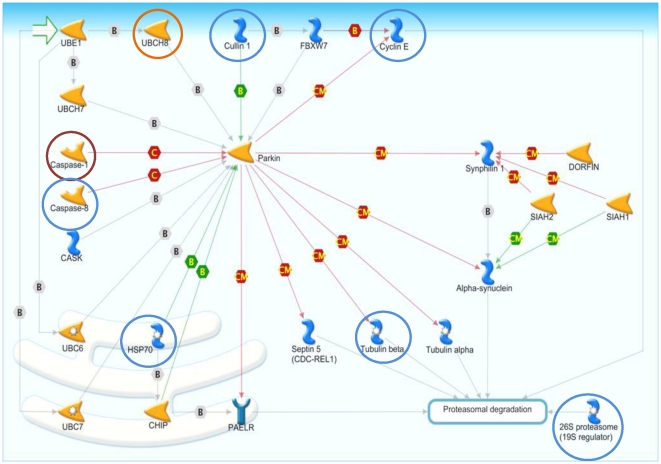
Parkin-Ubiquitin Proteasomal System pathway with viral frequency. Viral frequencies superimposed for each of most frequently differentially expressed proteins, where red circles are differential expression of genes by 7 viruses, orange circles are differential expression of genes by at least 6 viruses, and blue circles are differential expression of genes by 5 viruses. See MetaCore website at http://www.genego.com/pdf/MC_legend.pdf for figure legend and Table S4 for pathway map gene products' corresponding HUGO gene names.

There were 67 enriched pathways in which all seven respiratory viruses were represented by at least one comparison group (Table S3). The list is ranked first by the viral frequency, followed by the sum of the normalized viral expression (NVE) for each pathway. Also shown are the differentially expressed as well as the total number of network objects across all 28 comparisons. The top 20 enriched pathways are listed in Table 2 along with the percentage and names of the differentially expressed genes with a viral frequency of at least five in each pathway. Of these, the top five pathways were chosen for further analysis and mapping. These pathways are epidermal growth factor receptor (EGFR) signaling, CD40 signaling, interferon-gamma (IFNG) signaling, histamine receptor H1 (HRH1) signaling, and interleukin-17 (IL17) signaling (Figures S5. S6, S7, S8, S9; Table S4). Additionally, the Parkin-Ubiquitin Proteasomal System (Parkin-UPS) pathway was chosen for further analysis because it has not been previously associated with the innate immunity and might be an interesting new mechanism of host response to respiratory viral infection (Figure 3).

**Table 2:** Top twenty pathways with highly expressed gene percentages and names.

Pathway	% genes with VF^1^ ≥5	Names of genes with VF ≥5
EGFR signaling	26	JUN, MYC, NFKBIA, STAT1, FOS, JAK2, HBEGF, DUSP1, DUSP4, PTK2, GSK3B, MMP9, NFKB2, PIK3R1, PRKCA, SOS2, TGFA
CD40 signaling	31	IL8, JUN, NFKBIA, TNFAIP3, CCL2, FAS, IL6, IRF1, JAK2, PTGS2, TRAF1, CCND2, CD86, ICAM1, LYN, MAP2K3, MAP3K14, MAPK14, NFKB2, PIK3R1, TP53, TRAF5
IFNG signaling	24	MYC, STAT1, CDKN1A, EIF2AK2, IRF1, JAK2, SOCS1, STAT2, CAMK2G, CEBPB, ICAM1, MAPK14, PIK3R1, PRKCA, PTPN11
HRH1 signaling	25	IL8, JUN, NFKBIA, FOS, IL6, TNF, CSF2, F3, GNAQ, GNB4, GNG12, ICAM1, MAPK14, MMP9, PLA2G4C, PLCB1, PPP3CA, PRKCA
IL17 signaling	31	CEBPD, IL8, JUN, NFKBIA, CCL2, CCL20, CXCL1, FOS, IL6, JAK2, PTGS2, CEBPB, CSF2, GSK3B, ICAM1, IL1B, MAP2K3, MAP3K14, MAPK14, MMP9, NFKB2, PIK3R1
CSF2 signaling	25	EGR1, MYC, NFKBIA, CCL2, FOS, JAK2, MCL1, CSF2, LYN, NFKB2, PIK3R1, PIM1, PTPN11, SOS2
IL1 signaling	36	IL8, JUN, NFKBIA, STAT1, FOS, IL6, IRF1, PTGS2, TNF, EDN1, F3, FOSB, FOSL1, FOSL2, IL1B, IL1RAP, JUNB, MAP2K3, MAPK14, SERPINE1
CCR5 signaling	19	JUN, STAT1, FOS, JAK2, CCL4, CCL5, GNAQ, GNB4, GNG12, MAP2K3, MAPK14, PLCB1, PPP3CA, PRKCA, TIAM1
Chemokines-adhesion	16	CCR1, CXCL1, IL8, JUN, MYC, THBS1, CCL2, PLAUR, ARPC1B, CD47, FLNA, GNB4, GNG12, GSK3B, ITGA6, NFKB2, PIK3R1, PLAT, PLAU, PTK2, RAP1GAP, SERPINE1, SOS2, WASL
Cytoskeleton via TGF, WNT	10	JUN, MYC, CDKN1A, FOXO3, PLAUR, SERPING1, ARPC1B, CDKN2B, GSK3B, MAP2K3, MAPK14, PIK3R1, PLAT, PLAU, PTK2, SERPINE1, SOS2, TGFBR2, WASL
IL15 signaling	19	IL15, IL8, MYC, NFKBIA, FOS, IL6, MCL1, IL15RA, MAPK14, PIK3R1, PLCB1, PTK2, SOS2
IL22 signaling	17	JUN, MYC, STAT1, FOS, JAK2, MCL1, CD86, HLA-DOB, MAPK14, SOCS3, STAT4
Histamine-dendritic signaling	16	IL6, IL8, CCL2, CCL5, CD86, CREM, GNAQ, GNB4, GNG12, IL1B, IRF8, PRKACB, TNF
GnRH signaling	12	ATF3, DUSP1, EGR1, FOS, JUN, DUSP4, FOSL1, FOSL2, GNAQ, MAPK14, PRKACB
Prolactin receptor signaling	24	JUN, MYC, STAT1, IRF1, JAK2, NMI, NR3C1, OAS1, SOCS1, CEBPB, IRS1, PIK3R1, PTPN11, SOCS3, SOS2
JUN mediated metabolism	22	JUN, CDKN1A, FAS, FOS, PLAUR, TNFAIP6, FOSB, FOSL1, FOSL2, JUNB
Parkin-UPS	18	CASP1, CASP8, CCNE1, CUL1, HSPA1A, HSPA1B, HSPA1L, HSPA4, HSPA6, HSPA8, PSMD10, TUBB3, UBE2L6
Cytoskeleton remodeling	9	MYC, CDKN1A, JUN, PLAUR, ITGA6, MAPK14, MYH10, PIK3R1, PLAT, PLAU, PTK2, SERPING1, TGFBR2, WASL
IL2 signaling	15	MYC, EGR1, FOS, JUN, NFKBIA, FOSL1, FOSL2, NFKB2, PIK3R1, SOCS3
Gastrin signaling	19	CXCL1, CXCL2, FOS, HBEGF, IL8, IRS1, JUN, NFKBIA, GNAQ, MAPK14, MEF2A, PIK3R1, PTGS2, PTK2

1 VF = Viral Frequency.

The NVEs for differentially expressed genes with frequencies of at least six viruses are shown in Table 3 along with their associated pathways. The list is ranked by the greatest viral frequency, and then by number of pathways in which the gene is differentially expressed. The NVE values for all genes, along with associated pathways, ranked by the greatest viral frequency, followed by the number of pathways in which the gene is differentially expressed are in Table S5. We ensured that the NVE was not bias toward any particular comparison group, and indeed no single dataset contributed to the overall NVE for any single virus (Table S2). Hierarchical clustering on the quantile normalized fold change values for all genes having expression values in at least 26 out of 28 comparisons (at least 90% comparisons) and significant in at least seven comparisons (Figure S3) as well as for genes with NVE of at least six viruses (Figure S4) did not reveal any dominant clustering by GSE or virus type. The most consistently up-regulated genes (up-regulated in at least six viruses and down-regulated no more than one virus) are: nuclear factor of kappa light polypeptide gene enhancer in B-cells inhibitor alpha (NFKBIA), tumor necrosis factor alpha-induced protein 3 (TNFAIP3), chemokine C-C motif ligand 2 (CCL2), interferon regulatory factor 1 (IRF1), prostaglandin-endoperoxide synthase 2 (PTGS2), chemokine C-C motif ligand 20 (CCL20), dual specificity phosphatase 1 (DUSP1), eukaryotic translation initiation factor 2-alpha kinase 2 (EIF2AK2), TNF receptor superfamily member 6 (FAS), suppressor of cytokine signaling 1 (SOCS1), TNF receptor-associated factor 1 (TRAF1), and ubiquitin-conjugating enzyme E2L 6 (UBE2L6). There were no consistently down-regulated mRNAs (down-regulated in at least six viruses and up-regulated in no more than one virus).

**Table 3:** Normalized viral expression and pathway inclusion grid for genes with viral frequency ≥6.

Gene Name^1^	CMV		CORON		COX		FLU		HRV		MPENU		RSV		Included in pathway					
	Down	Up	Down	Up	Down	Up	Down	Up	Down	Up	Down	Up	Down	Up	EGFR	HRH1	IFNG	IL17	CD40	Parkin
JUN	−2/5			3/4		1/3	−2/6		−1/2			5/5		1/3	X	X		X	X	
NFKBIA		1/5		3/4		1/3		1/6		2/2		4/5		1/3	X	X		X	X	
IL8	−2/5	1/5		2/4		1/3	−1/6	1/6		1/2		5/5		1/3		X		X	X	
MYC	−2/5		−1/4			2/3		1/6		1/2		3/5		1/3	X		X			
STAT1		5/5	−1/4	1/4	−1/3			4/6	−1/2	1/2		5/5	−1/3	1/3	X		X			
CEBPD	−1/5			1/4		1/3		1/6		1/2	−1/5	2/5		1/3				X		
TNFAIP3		3/5		2/4		1/3		1/6		2/2		5/5		1/3					X	
CASP1		4/5	−1/4	1/4	−1/3			2/6	−1/2	1/2		4/5		1/3						X
JAK2	−1/5	2/5		1/4				2/6		1/2		4/5	−1/3	1/3	X		X	X	X	
FOS	−3/5			3/4		2/3	−1/6				−1/5	3/5		1/3	X	X		X		
IL6	−1/5	4/5		1/4			−1/6	2/6		2/2		5/5		1/3		X		X	X	
CCL2		3/5				1/3		1/6		1/2	−1/5	4/5		1/3				X	X	
IRF1		5/5		1/4				2/6		1/2	−1/5	4/5		1/3			X		X	
PTGS2		3/5		1/4		1/3	−1/6	1/6		1/2		5/5						X	X	
CCL20		1/5		2/4			−1/6	2/6		2/2		3/5		1/3				X		
CDKN1A		2/5				1/3	−1/6	2/6		1/2	−1/5			1/3			X			
CXCL1	−1/5	1/5		2/4		1/3	−2/6			2/2		3/5						X		
DUSP1		1/5		3/4		2/3	−2/6	1/6				3/5		1/3	X					
DUSP4		1/5	−1/4			1/3		1/6			−1/5	3/5		1/3	X					
EIF2AK2		3/5				1/3		4/6		1/2		4/5		1/3			X			
FAS		5/5				1/3		1/6	−1/2	1/2		5/5		1/3					X	
HBEGF		3/5				2/3	−4/6			2/2		5/5		1/3	X					
SOCS1		3/5		1/4				3/6		2/2		4/5		1/3			X			
STAT2		4/5	−1/4	1/4			−1/6	2/6		1/2		4/5		1/3			X			
TNF	−1/5	4/5		1/4		1/3		2/6		2/2	−1/5	2/5				X				
TRAF1		3/5	−1/4	1/4		1/3		1/6		1/2		3/5							X	
UBE2L6		5/5		1/4				4/6		1/2		5/5		1/3						X

1 The first 8 genes are differentially expressed in seven viruses, and the remaining 19 genes are differentially expressed in six viruses.

We sought drug repurposing candidate targets from the top five enriched pathways and the Parkin-UPS pathway by searching the DrugBank database, version 3.0 (http://www.drugbank.ca/ accessed August 2011) [29]–[31], for drugs targeting any of the 67 differentially expressed genes with a viral frequency of at least five (Table S6). Of these, thirteen genes, or almost 20% of the original 67 genes, were associated with at least one approved small molecule or protein therapy. There genes were: prostaglandin-endoperoxide synthase 2 (PTGS2), TNF, matrix metallopeptidase 9 (MMP9), jun proto-oncogene (JUN), interleukin 1 beta (IL1B), CCL2, CD86, coagulation factor III (F3), phosphoinositide-3-kinase regulatory subunit 1 (PIK3R1), intercellular adhesion molecule 1 (ICAM1), nuclear factor of kappa light polypeptide gene enhancer in B-cells 2 (NFKB2), Caspase 1 (CASP1), and tubulin beta 3 (TUBB3). A selection of these genes, along with other characteristics to evaluate their potential as drug targets such as involvement in immune response [29]–[31], Jackson Laboratory knock-in/knock-out mouse (JAX) phenotype [32], approved or marketed small molecule drug or protein therapy, and current indications for that drug, are listed in Table 4. Note that the current indication may not be for the gene target listed. Mimosine (gene target: CCL2) and Glucosamine (gene targets: NFKB2 and MMP9) did not have a current indication, while the interactions of Natalizumab (gene target: ICAM1) and Gallium nitrate (gene target: ILB1) with their gene targets were unclear. Additionally, therapies associated with PTGS2 are cyclooxygenase (COX-2) inhibitors which have known side-effect issues thus were not explored further. Therefore, NFKB2, ICAM1 and PTGS2 were excluded from Table 4, leaving ten genes for potential drug repurposing. The potential cases for drug repurposing are discussed more in-depth for four targets; F3, IL1B, TNF and CASP1.

**Table 4:** Putative targets with associated drugs.

Gene^1^	Involvement in immunity [29]–[31]	JAX phenotype^3^	Drug Name	Current Indication^3^ [29]–[31], [103]
CCL2	activator^2^	not available	Danazol	endometriosis, benign breast disorders, angioedema
F3	activator	none	Coagulation factor VIIa	hemorrhagic complications in hemophilia A and B
PIK3R1	activator; used by viruses	abnormal humoral immune response & B cell physiology	Isoproterenol	mild/transient heart block; asthma and chronic bronchitis
CD86	activator^2^	abnormal humoral immune response; dec. T cell proliferation	Abatacept	RA; polyarticular JIA
			Antithymocyte globulin	renal transplant rejection
IL1B	activator	inc. susceptibility to bacterial infection	Minocycline	bacterial infections
			Canakinumab	CAPS
TNF	activator^2^	abnormal immune system physiology, inc. susceptibility to viral infection, inc./dec. susceptibility to bacterial infection	Infliximab	Crohn's disease; ulcerative colitis; RA, JIA & psoriatic arthritis; ankylosing spondylitis
			Pranlukast	reduces bronchospasm caused by allergic reaction
			Amrinone	congestive heart failure
			Etanercept, Adalimumab	RA; JIA (Etanercept); psoriatic arthritis (Adalimumab); ankylosing spondylitis; severe plaque psoriasis (Etanercept); Crohn's disease (Adalimumab)
			Thalidomide	multiple myeloma and erythema nodosum leprosum
			Chloroquine	malaria; RA
			Amrinone	congestive heart failure
			Clenbuterol	bronchodilator for asthma attacks
MMP9	used by viruses	abnormal histamine physiology	Marimastat	cancer
			Minocycline	bacterial infections
			Captopril	renovascular hypertension; congestive heart failure; left ventricular dysfunction; nephropathy
JUN	activator^2^	none	Irbesartan	hypertension; nephropathy in type 2 diabetic patients
			Arsenic trioxide	acute promyelocytic leukemia
CASP1	activator^2^	dec. inflammatory response, inc./dec. susceptibility to bacterial infection	Minocycline	bacterial infections
TUBB3	used by viruses	not available	Ixabepilone	breast cancer

1 CASP1 and TUBB3 are members of Parkin-UPS pathway.

2 Strongly associated with innate immune response activation.

3 RA = rheumatoid arthritis; JIA = juvenile idiopathic arthritis; CAPS = cryopyrin-associated periodic syndromes; inc. = increased; dec. = decreased.

## Discussion

### Analysis of mRNA Microarray Datasets

Our study used a systematic process to minimize potential technical noise that could have arisen from our comparative analysis of fifteen unique datasets from nine different cell types, and seven different array platforms. These measures included candidate dataset filtering followed by QC, differential gene expression and pathway enrichment analyses. A total of 14 out of 42, about one third of the total comparisons, were removed as a result of this filtering process, which is indicative of our conservative analysis approach. We had previously used large-scale and merged-SAM analyses in integrating large-scale microarray datasets involving cancer tissues from multiple laboratories [33], [34]. However, the small sample size datasets used in the present study required a more rigorous methodology to identify data outliers.

To our knowledge the QC analysis performed with each GEO dataset is unique to this study. Although no dataset was completely disregarded after QC analysis, some samples were clear outliers, thus potentially skewing the data. Kauffmann and Huber have demonstrated improvements in signal-to-noise ratios after performing post normalization QC analysis to remove array outliers within an experiment [35]. Those authors used MA-plot and box-plots of the log-ratios to determine outliers instead of MAD scores, PCA and pair-wise correlations employed in this study. Fundamentally, the concept of data improvement after outlier removal applies regardless of the QC analysis approach.

### Pathways Modulated by Virus Infection

Despite the diverse nature of the microarray data analyzed here, we found a large overlap between comparison groups in significant pathways, especially the immune system. Of the top twenty enriched pathways, eighteen are associated with immune response (Table 2). For example, EGFR signaling is known to be activated during infection by respiratory viruses FLU [36] and ENTERO [37], [38]. CD40 signaling is associated with CORON [39], RSV [40], and the general immune response [41]. Interferon gamma (IFNG) signaling is initiated by FLU [42] and RSV [43], while interleukin 1 signaling is stimulated by FLU [42]. As components of the general immune response, interferon and interleukin pathways are activated by infectious agents such as hepatitis C virus (HCV), HIV and tuberculosis as well as chronic diseases like Crohn's disease, diabetes, and metastatic melanoma [44], [45]. The overall relationships between the transitory host immunity response launched by pathogenic infections versus that seen in chronic autoimmune and neurodegenerative diseases are complex and an intense area of investigation [46]. In addition, there are considerations about subtle shifts in gene function roles in different cell tissue types amongst the various diseases. Thus, we are cautious about any linkages between pathways involved in infections and those of chronic diseases as implied by our analysis without further validation studies.

### Potential Role of Parkin-UPS Pathway in Viral Infection

Parkin (PARK2) is an E3-ubiqutin ligase associated with the progression of the neurodegenerative disorder Parkinson's disease. [47]. As a central hub protein in the Parkin-UPS pathway, PARK2 ubiquinates proteins encoded by septin 5 (SEPT5) [48], tubulin alpha and beta [49], and the glycosylated form of synuclein, alpha (SNCA) [50] for degradation by the 26S proteasome. PARK2 also ubiquinates synuclein, alpha interacting protein (SNCAIP) for regulation of SNCA [51], interacts with STIP1 homology and U-box containing protein 1 E3 ubiquitin protein ligase (STUB1) to enhance ubiquitination of G protein-coupled receptor 37 (GPR37), [52] (which associates with F-box and WD repeat domain containing 7 (FBXW7)), and cullin 1 (CUL1) to ubiquitinate cyclin E [53]. PARK2 is deactivated by protolytic cleavage by CASP1 and Caspase 8 (CASP 8) [54] and can be activated by either heat shock protein 70kD (HSPA4) or STUB1 [52].

The Parkin-UPS pathway is not commonly associated with general immune response to viral infection. However, other ubiquitylation proteins, such as ISG15, are known to play roles in host defense [55], [56]. Associations between influenza infection and neuroinflammation in early onset autosomal recessive Parkinson's disease have been recently suggested [57]–[59]. At least one factor in the progression of Parkinson's disease is the formation of neuotoxic Lewy bodies due to increases in SNCA. Increases in SNCA are believed to be the result of loss-of-function mutations in PARK2 which cause disruptions in the protein's localization and solubility [60]–[62]. Polymorphisms in the gene PARK2 have also been associated with susceptibility to infectious diseases such as leprosy, typhoid fever and paratyphoid fever, although the exact mechanism is still unclear [63], [64]. Jang *et al.* observed activation of SNCA in mouse nervous tissue long after pathogenic H5N1 FLU infection where the increased levels of SNCA mirror those found in Parkinson's disease [57]. Similarly, recent findings from Rohn and Catlin indicate FLU as a potential causative factor for Parkinson's disease [58]. Indeed, links between FLU and other neurodegenerative diseases have been suggested, and these include seizures, transverse myelitis, expressive aphasia, syncope, encephalitis, neuromyelitis optica, and central nervous system disease in general [65]–[67].

PARK2 itself has a low signal at the mRNA level which might be due to its significant regulation by post-translation processes [52], [54]. Further studies are needed to determine the mechanism by which viruses modulate the Parkin-UPS pathway during infection.

### Drug Repurposing Against Respiratory Viruses

Our analysis suggests several potential repurposing opportunities for launched drugs against host-viral targets (Table 4). This assumption is based on the occurrence of genes that are differentially expressed in infection models for at least five of the seven respiratory viruses, have involvement in a number of relevant pathways related to host immune response, and encode for known drug targets. The drugs associated with this gene list do not have current indications as anti-viral therapies, although Pranlukast and Clenbuterol are prescribed for relief of lung disorders such as bronchospasm after allergic reactions and asthma bronchoconstriction during asthma attacks, respectively. Also, Minocycline, sometimes called Minocin, is a broad-spectrum tetracycline antibiotic as well as a caspase 1 (CASP1) inhibitor while Chloroquine is a well-known anti- malaria drug [29]–[31]. In fact, eight of the ten drug repurposing gene targets are involved in activation of the innate immune response, while the remaining two have some evidence of virus modulation. Potential drug repurposing opportunities for F3, IL1B, TNF, and MMP9, as well as the Parkin-UPS pathway gene product CASP1, are discussed below.

#### Coagulation Factor III (F3)

F3 normally binds to the native cofactor VII or VIIa to induce the blood coagulation cascade. Treatment with recombinant coagulation factor VIIa promotes blood coagulation in hemophiliacs [29]–[31]. Esmon et al. [68] suggest that coagulation could be used therapeutically to modulate inflammation responses and vice versa, but also caution about the danger of increased incidence of thrombosis. The consistent up-regulation of F3 across five viruses suggests that the immune-coagulation axis is already initiated and supplemental F3 activation may cause thrombosis complications. Further study is needed to develop therapeutics that could balance between innate immune response triggered by coagulation factor VIIa therapy and stabilization of the antithrombotic state.

#### Interleukin 1 beta (IL1B)

IL1B is a cytokine involved in inflammatory response, cell proliferation, differentiation, and apoptosis. IL1B is specifically cleaved into its active form by the protease CASP1 after which it activates the NLRP3 inflammasome [29]–[31], [69]. Indeed, IL1B is consistently up regulated across CMV, FLU, HRV, MPENU and RSV which likely correlates with inflammasome activation. However, over-expression of IL1B causes multiple inflammatory disorders [69]. Antagonists or neutralizers of IL1B, such as Canakinumab, could potentially reduce inflammation damage associated with viral infection.

#### Tumor Necrosis Factor (TNF)

TNF has a wide range of biological functions including modulation of immune response to pathogen assault. Mouse TNF knock-out phenotypes include abnormal immune system physiology, increased susceptibility to viral infection, and both increased and decreased susceptibility to bacterial infection [29]–[31]. In our study, TNF is mostly up regulated in infections by CMV, CORON, COX, and FLU but directionally ambiguous for MPNEU and not expressed under RSV. While total disruption of TNF function would be deleterious to the host, there are instances where partial TNF inhibition provides a clinical benefit in patients with viral complications [70], [71].

Pranlukast is a cysteinyl leukotriene receptor-1 antagonist that reduces bronchospasm caused by an allergic reaction, usually with asthmatic individuals. This drug inhibits TNF-alpha by blocking macrophage cysteinyl leukotriene 1 (cysLTC4, D4) receptors [72] or suppression of NF-kappa B activation [73]. Pranlukast has been recently shown to be beneficial not only in cases of respiratory syncytial virus postbronchiolitis, but also in a wide variety of other diseases with strong inflammatory complications such as cystic fibrosis, cancer, atherosclerosis, eosinophils cystitis, otitis media, capsular contracture, and eosinophilic gastrointestinal disorders [71].

Amrinone is a type 3 pyridine phosphodiesterase inhibitor used in the treatment of congestive heart failure and is an inhibitor of TNF [74]. Phosphodiesterase inhibitors have been shown to alter immune response [75]–[78] and, in one case, specifically through TNF [79]. Amrinone is known to modulate pro- and anti-inflammatory factors in endotoxin-stimulated cells [80]. Type 4 phosphodiesterase inhibitors have been used to treat RSV-induced airway hyper-responsiveness and lung eosinophilia [81]. Therefore, indirect evidence suggests that Armirone may be beneficial in respiratory viral infection situations by inhibiting TNF via type 4 phosphodiesterase, although this has yet to be seen in clinical studies.

#### Matrix Metallopeptidase 9 (MMP9)

MMP9 encodes a matrix metallopeptidase that degrades type IV and V collagens, and is implicated in arthritis and metastasis [29]–[31]. We can only speculate on the role MMP9 plays in infection. Our analysis finds the gene to be up-regulated for three viruses while down-regulated for two different viruses. In other studies, MMP9 has been observed to be up-regulated after exposure to double stranded RNA and is important to airway injury [82], specifically by RSV [83]. MMP9 expression is induced by IL1B [84] which, as mentioned above, is an activator of the NLRP3 inflammasome [85]. MMP9 inhibitors such as Marimastat, Minocycline or Captopril, could be beneficial assuming that the protein is co-opted by the infecting virus for tissue remodeling. Blocking MMP9 may also reduce inflammatory damage by down-regulating the inflammasome.

#### Caspase 1 (CASP1)

In the case of the Parkin-UPS pathway, inhibiting tubulin-beta formation may reduce viral proliferation given that FLU utilize acetylated tubulin for protein trafficking [86] and increases in neuronal class III TUBB occur after COX infection [87]. A CASP1 inhibitor such as Minocycline could be used to increase PARK2 ubiquitinase activity, in turn decreasing the TUBA or TUBB availability.

As mentioned above, CASP1 is a component of the NLRP3 inflammasome, activating the precursor to IL1B [69]. Therefore, a CASP1 inhibitor would have an antagonist relationship with IL1B, hence the inflammasome. Further, CASP1 inhibitors would be agonists for PARK2, thereby reducing accumulation of SNCA. In this regard, CASP1 inhibitors may not only prevent unnecessary NLRP3 inflammasome activation via ILB1, but may also reduce accumulation of neurotoxic Lewy bodies through activation of PARK2.

However, caspases are not specific to the Parkin-UPS pathway and inhibition in this regard may result in toxicity or other complications [88]. Additionally, mouse JAX phenotypes for CASP1 show both increased and decreased susceptibility to bacterial infection, as well as decreased inflammatory response. While CASP1 inhibition may prove beneficial in terms of increasing inflammatory responses, it is ambiguous in terms of benefit for bacterial infections. In our analysis, the expression of CASP1 and TUBB3 is also somewhat variable across virus types. Therefore, more study is needed specifically on the role of caspase and tubulin in host response to respiratory virus infection.

### Future Directions

Modulation of any human host pathway for the treatment of viral infections has potential drawbacks with respect to toxicity and other side-effects. For example, although interferon is widely used to help combat viral pathogens, the treatment is known to cause an array of side-effects related to toxicity including confusion, lethargy, impaired mental status, numbness, tingling, fevers, chills, headaches, anorexia and sepsis [89], [90]. Another caveat is that some proteins are beneficial if up-regulated during initial viral infection but have detrimental effects if over-activated for prolonged periods. Thus determining the desired mechanism and direction of therapeutic intervention requires careful study. Although targeting host-pathogen interactions is a challenging therapeutic approach, there are considerable upside benefits with respect to overcoming pathogen-mediated drug resistance and the capability of treating multiple, co-infecting pathogens. Our study suggests several potential human-host proteins that could be targets of future therapeutics as well as some possible drug candidates for further investigations of repurposing against respiratory virus infections.

## Materials and Methods

### Data Sources for Human mRNA Datasets

The National Center for Biotechnology Information's GEO database (http://www.ncbi.nlm.nih.gov/geo/ (accessed between January and July 2011) was searched for human mRNA datasets for twelve respiratory viruses. These viruses were the double stranded DNA viruses *Herpesviridae*, human cytomegalovirus and *Adenoviridae* Adenovirus; the positive sense single stranded RNA viruses *Coronaviridae* SARS-Coronavirus, *Picornaviridae* Coxsackievirus, *Picornaviridae* Human Rhinovirus, *Picornaviridae* Echovirus, *and Picornaviridae* Enterovirus; and the negative sense single stranded RNA viruses *Orthomyxoviridae* Influenza A virus, *Paramyxoviridae* Metapenumonia virus, *Paramyxoviridae* Respiratory syncytial virus, *Bunyaviridae* Hantavirus and Sin nombre virus. Subsequent filtering steps (Figure 1) reduced the number of viruses with suitable datasets to seven species (Table 1).

All analyzed GEO datasets contain at least one “treatment group” and “control group”. “Treatment” was the experimental variable under study, usually a virus type, strain, or time point. “Group” was a collection of individual “samples”, or replicates, each of which originates from their own microarray chip. “Comparison group” was the treatment group compared to a control group. A particular dataset may have more than one comparison group. All criteria for dataset inclusion in the final analysis were chosen prior to the analysis.

### Dataset Selection and Quality Control Processes

Dataset candidacy filtering consisted of four criteria: 1) the dataset must contain at least 3 samples per treatment or control group because a sample size any less would mean a loss in statistical power for subsequent analysis; 2) the microarray platform must be supported by either Affymetrix, Agilent or Illumina due to probe mapping abilities of the software used in subsequent analysis; 3) each gene expression profile had to be derived from human cells and probed using a human-based genome microarray platform and not other species; and 4) the dataset must contain at least one wild-type infection treatment group (i.e., unmodified virus strain or infectivity mechanism) and at least one healthy control group (i.e., no genetic or media modifications such as gene knock outs or inhibitors, respectively).

Prior to quality control (QC) analysis, we pre-screened and pre-processed each dataset. Normalized raw data and the study design table were imported from the GEO databases (The data was assumed to be normalized by robust multi-array average, but in some cases the published study used an alternative normalization method). Where appropriate, the intensity values were log_2_ transformation. Various experimental parameters such as time point, virus strain and number of replicates were extracted from the study design tables. Samples irrelevant to the main study design were marked for segregation or exclusion from our downstream analysis, but not excluded from quality assessment. These were classified as “failing to meet treatment specification” at the candidate filtering step. Studies that had a large number of missing intensity values (over 10%) were annotated and flagged.

The QC analysis assessed each sample in the dataset for kernel density, PCA, MAD, and pair-wise Pearson correlation such that: 1) the kernel density was normally distributed; 2) after PCA values were within the Hotelling T2 alpha level threshold of 0.05 [91]–[93]; 3) MAD score scores were in the range of +3 to −3 with no outliers [94]; and 4) inner-treatment group pair wise correlations for samples derived from a single cell were ≥0.97 or ≥0.90 if taken from individual donors [94]. Figures were created using Array Studio software, version 4.1. (Omicsoft Corporation, Research Triangle Park, NC, USA [95]). During subsequent analysis, each comparison group was treated separately, regardless of dataset origination, in order to gain a wider, less bias view of representative genes and pathways.

### mRNA Array Expression and Pathway Analysis

Once a comparison group passed the QC analysis filters, LSM values were calculated for each probe using Array Studio in order to reduce the number of false positives due to low probe intensity values. Probes within each of the filtered datasets were tested for biological and statistical relevance using the Array Studio implementation of fold change and statistical models, respectively. Specifically, to determine a probe's fold change expression when compared to control, the geometric mean of each probe's log_2_ transformed intensity value within a treatment was generated, and then normalized to the corresponding control group's geometric mean. The treatment versus control data were fitted to a general linear model, and associated p-values for each probe were calculated using a modified t-test [96]. Thus, to be considered differentially expressed, each probe within a comparison group must have a p-value <0.05 after general linear model test and a fold change in either direction of 1.5.

To visualize a comparison group's significance and fold change, volcano plots were generated using Array Studio of a probe's −log(p-value) versus its transformed fold change (FC) value according to the following piece-wise function:
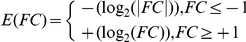



The differentially expressed probes were mapped to their corresponding genes using MetaCore/MetaBase (GeneGo), a software/database package that creates biological pathways and networks from gene lists (database accessed June 2011) [97], [98]. If more than one probe mapped to a gene, the probe with the highest magnitude fold change value was used for that gene. Thus, the mapped differentially expressed probe list became the differentially expressed gene list for each comparison group.

The differentially expressed gene lists from each comparison group were analyzed for enriched pathways using GeneGo. A p-value for each of the 658 pathway maps in the MetaBase were generated for each comparison group using a hypergeometric test [99]. In order for a pathway to be considered enriched, each comparison group must contain pathways that have a p-value <0.01 and occur in >5% of the total studies. The enriched pathway list was ranked by its viral frequency, which is defined by the number of viruses represented by at least one comparison group, and then by the sum of Normalized Viral Expression or NVE for each enriched pathway. The NVE for each pathway was calculated using the number of comparisons containing significant pathways within a virus type relative to the number of comparisons within that virus type. For example, if one out of four FLU comparisons for pathway *A* were significant, the NVE for FLU would be 1/4. Ranking the pathways in this fashion resulted in a clearer determination of pathways shared across multiple viruses, irrespective of time, strain type, or number of comparison groups.

After examining the ranked pathway list described above, the top five significant pathways and an additional pathway representing a unique mechanism were further analyzed. With each map, the proteins were labeled according to the number of viruses in which the transcript was differentially expressed thus yielding the viral frequency for that protein. In cases where a protein complex was made up of subunits, the greatest magnitude fold change value for any subunit was chosen to represent the entire complex. GeneGo was used for the visualization of this pathway map.

Similar to the pathway NVE, the NVE for each gene within these six chosen pathways was calculated using the number of comparisons containing either up or down regulated genes for each protein within a virus type relative to the number of comparisons within that virus type. For example, if two out of three RSV comparisons for gene X were up-regulated, gene X's NVE for RSV would be 2/3.

We performed complete linkage and correlation distance hierarchical clustering using ArrayStudio on quantile normalized fold change values to determine the separation qualities of the analyzed data [100]. Clustering was performed on genes that had expression values for at least 90% of the total number of comparisons. We used the Matlab function ‘knnimpute’ to impute missing fold change values using k-Nearest Neighbors estimation (MATLAB version 7.11 (R2010b), Mathworks, Cambridge MA, 2010) [101], [102].

### Drug Repurposing

Approved or marketed small molecule and protein therapeutics for each of the differentially expressed proteins modulated by 5 or more respiratory viruses were obtained from the DrugBank database, version 3.0 (http://www.drugbank.ca/ accessed August 2011) [29]–[31]. We only considered those drugs that were launched products with experimental and clinical evidence of direct interaction with gene product in question. The therapy's interaction with the target and approved indication were identified using a combination of DrugBank, the drug manufacturer's information page, and the National Center for Biotechnology Information's PubChem (http://pubchem.ncbi.nlm.nih.gov/ accessed September 2011) [103] and Gene (http://www.ncbi.nlm.nih.gov/gene/ accessed September 2011) databases. Supplemental evidence of mechanism of action was obtained from immune or infection-related Jackson Laboratory knock-in/knock-out mouse (JAX) phenotype (http://www.jax.org/ accessed September 2011) [32].

## Supporting Information

Figure S1 **Sample of quality analysis for subset of GSE17156: RSV treatment and control groups using MAD score and correlations.** MAD score plot as a function of time point a) before and b) after removal of samples GSM429279 and GSM429252; Baseline group correlation heat map c) before and d) after removal of samples GSM429279 and GSM429252 (not shown): white blocks indicate pair-wise Pearson correlation below 0.97, dark blue indicate perfect (1.00) pair-wise Pearson correlation.
(TIF)

Figure S2 **Volcano plot of a probe differential expression analysis for the RSV symptomatic treatment from GSE17156.** Each point on the figure represents an individual mRNA array chip probe. Horizontal axis is estimate and vertical axis is −log(p-value). Red lines dictate threshold cutoffs of p-value 0.05 (−log (p-value)≈1.3) and fold change +/−1.5 (estimate≈+/−0.58).
(TIF)

Figure S3 **Hierarchical clustering of the fold change values on genes differentially expressed in at least 7 comparisons.** The horizontal axis contains each of the 28 different comparisons labeled by virus, GSE and time point. The vertical axis shows clustering of 1,274 genes that are differentially expressed in at least 7 of the 28 comparisons and have an expression value in at least 26 comparisons. Yellow indicates a fold change value of 3.0 or greater; blue indicates a fold change value of −3.0 or less; white indicates a fold change value of 0.0.
(TIF)

Figure S4 **Hierarchical clustering of the fold change values on genes from top pathways with an NVE of at least 6 viruses (** **Table 3** **).** The horizontal axis contains each of the 28 different comparisons labeled by virus, GSE and time point. The vertical axis shows the clustering of 27 genes from the top five and Parkin-UPS pathways that have an NVE of at least 6 and have an expression value in at least 26 comparisons. For genes present in more than one of the five pathways, the number of participating pathways is indicated by the count of “*” before the gene name. Color scheme is as described for Figure S3.
(TIF)

Figure S5 **Epidermal Growth Factor Receptor signaling pathway with viral frequency.** Viral frequencies superimposed for each of most frequently differentially expressed proteins, where red circles are differential expression of genes by 7 viruses, orange circles are differential expression of genes by at least 6 viruses, and blue circles are differential expression of genes by 5 viruses. See MetaCore website at http://www.genego.com/pdf/MC_legend.pdf for figure legend and Table S4 for pathway map gene products' corresponding HUGO gene names.
(TIF)

Figure S6 **CD40 signaling pathway with viral frequency.** Viral frequencies superimposed for each of most frequently differentially expressed proteins, where red circles are differential expression of genes by 7 viruses, orange circles are differential expression of genes by at least 6 viruses, and blue circles are differential expression of genes by 5 viruses. See MetaCore website at http://www.genego.com/pdf/MC_legend.pdf for figure legend and Table S4 for pathway map gene products' corresponding HUGO gene names.
(TIF)

Figure S7 **Interferon-gamma signaling pathway with viral frequency.** Viral frequencies superimposed for each of most frequently differentially expressed proteins, where red circles are differential expression of genes by 7 viruses, orange circles are differential expression of genes by at least 6 viruses, and blue circles are differential expression of genes by 5 viruses. See MetaCore website at http://www.genego.com/pdf/MC_legend.pdf for figure legend and Table S4 for pathway map gene products' corresponding HUGO gene names.
(TIF)

Figure S8 **Histamine Receptor H1 signaling pathway with viral frequency.** Viral frequencies superimposed for each of most frequently differentially expressed proteins, where red circles are differential expression of genes by 7 viruses, orange circles are differential expression of genes by at least 6 viruses, and blue circles are differential expression of genes by 5 viruses. See MetaCore website at http://www.genego.com/pdf/MC_legend.pdf for figure legend and Table S4 for pathway map gene products' corresponding HUGO gene names.
(TIF)

Figure S9 **Interleukin-17 signaling pathway with viral frequency.** Viral frequencies superimposed for each of most frequently differentially expressed proteins, where red circles are differential expression of genes by 7 viruses, orange circles are differential expression of genes by at least 6 viruses, and blue circles are differential expression of genes by 5 viruses. See MetaCore website at http://www.genego.com/pdf/MC_legend.pdf for figure legend and Table S4 for pathway map gene products' corresponding HUGO gene names.
(TIF)

Table S1 **GSM (GEO Sample) annotation for all studies considered.**
(XLSX)

Table S2 **Gene list of fold change, p-value and passes LSM threshold for the 28 analyzed comparisons.**
(XLSX)

Table S3 **Results of pathway enrichment analysis.**
(XLSX)

Table S4 **Gene symbol-network object name mapping.**
(XLSX)

Table S5 **Normalized viral expression and pathway inclusion grid.**
(XLSX)

Table S6 **Number of experimental and marketed drugs associated with the top genes from analyzed pathways.**
(XLSX)
