# Supplementary figures and images for: Identification of Common Biological Pathways and Drug Targets Across Multiple Respiratory Viruses Based on Human Host Gene Expression Analysis
Source: PLoS One. 2012 Mar 14;7(3):e33174. doi: 10.1371/journal.pone.0033174 (PMC3303816; doi:10.1371/journal.pone.0033174)

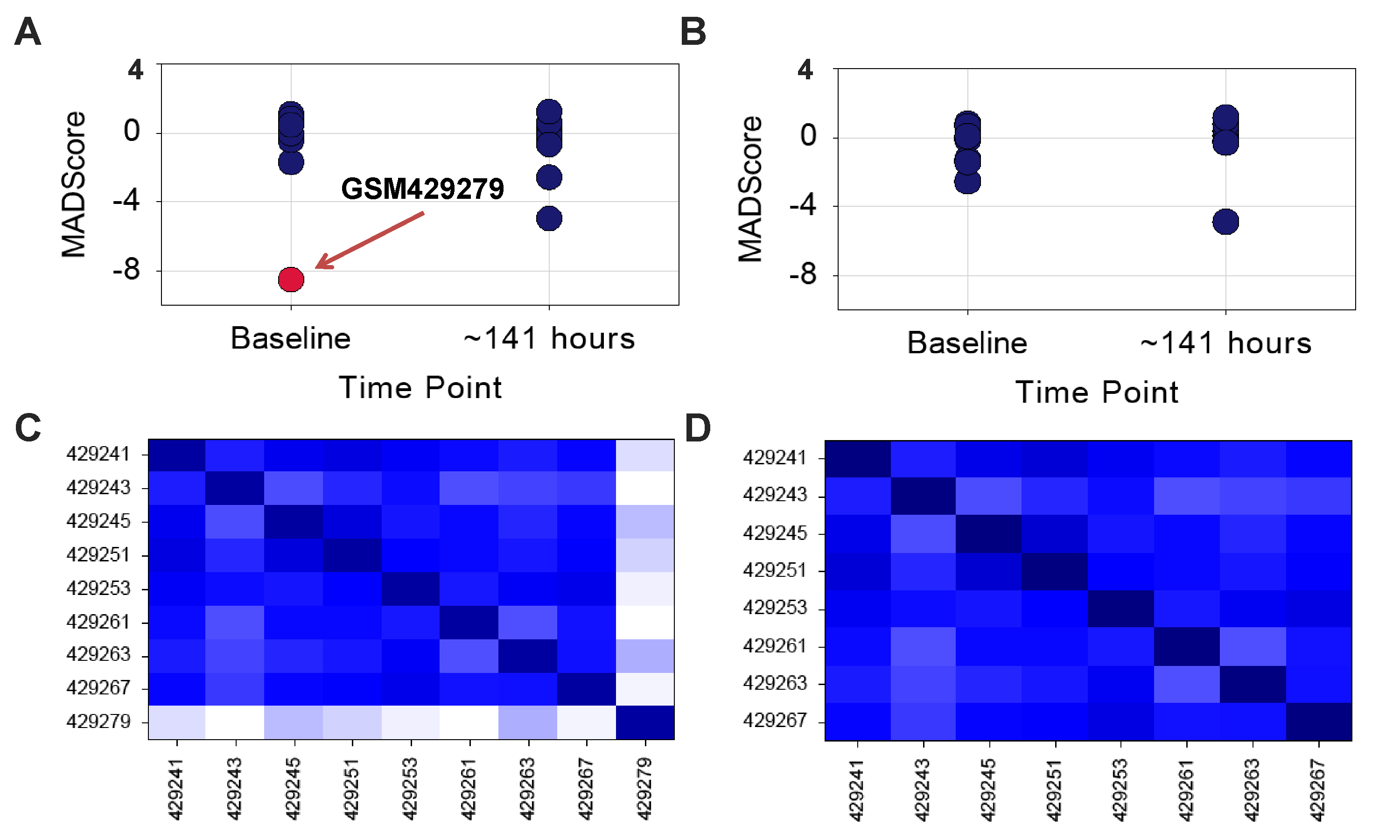

Supplement: Figure S1 — Sample of quality analysis for subset of GSE17156: RSV treatment and control groups using MAD score and correlations. MAD score plot as a function of time point a) before and b) after removal of samples GSM429279 and GSM429252; Baseline group correlation heat map c) before and d) after removal of samples GSM429279 and GSM429252 (not shown): white blocks indicate pair-wise Pearson correlation below 0.97, dark blue indicate perfect (1.00) pair-wise Pearson correlation. (TIF) [file pone.0033174.s001.tif]

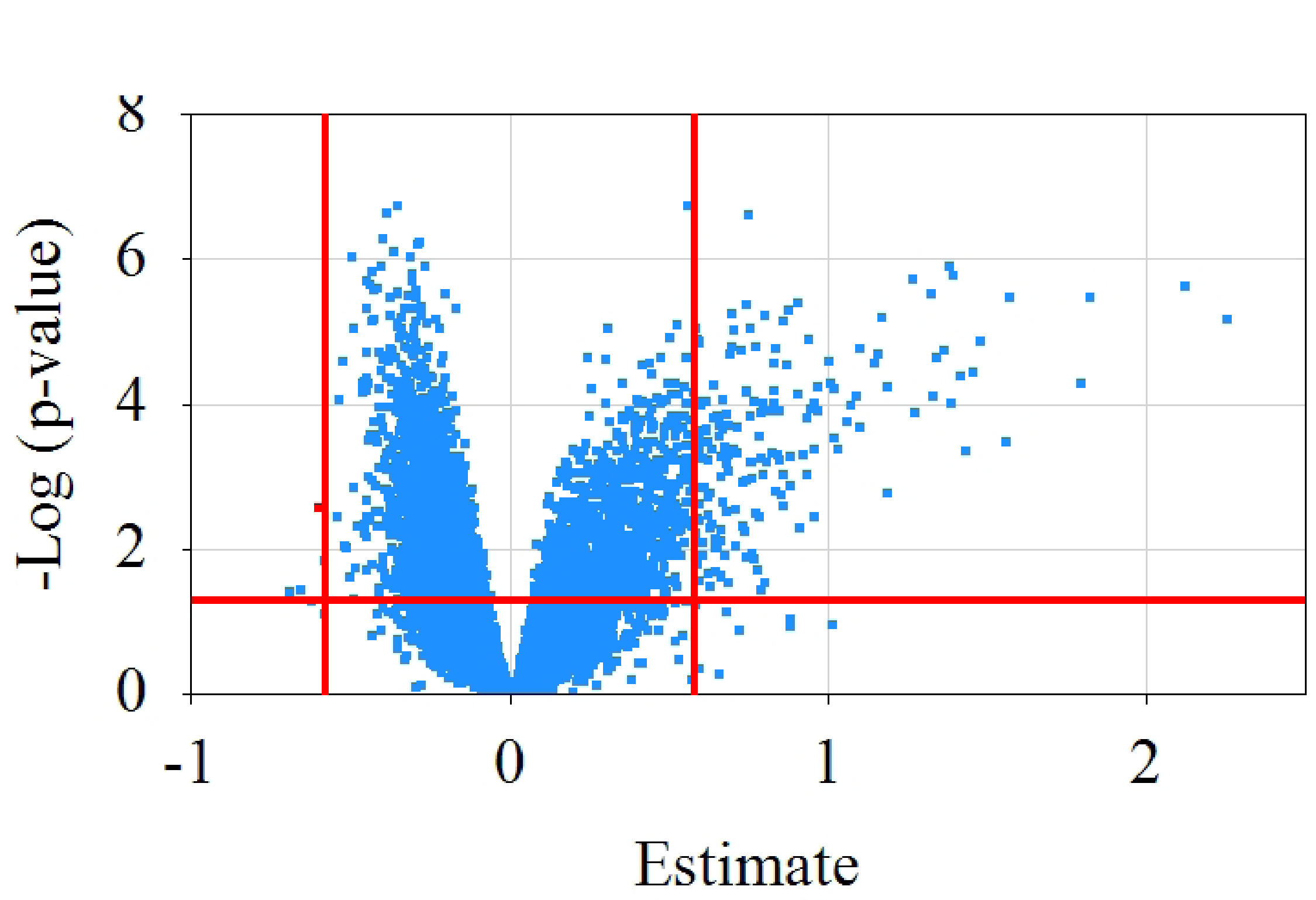

Supplement: Figure S2 — Volcano plot of a probe differential expression analysis for the RSV symptomatic treatment from GSE17156. Each point on the figure represents an individual mRNA array chip probe. Horizontal axis is estimate and vertical axis is −log(p-value). Red lines dictate threshold cutoffs of p-value 0.05 (−log (p-value)≈1.3) and fold change +/−1.5 (estimate≈+/−0.58). (TIF) [file pone.0033174.s002.tif]

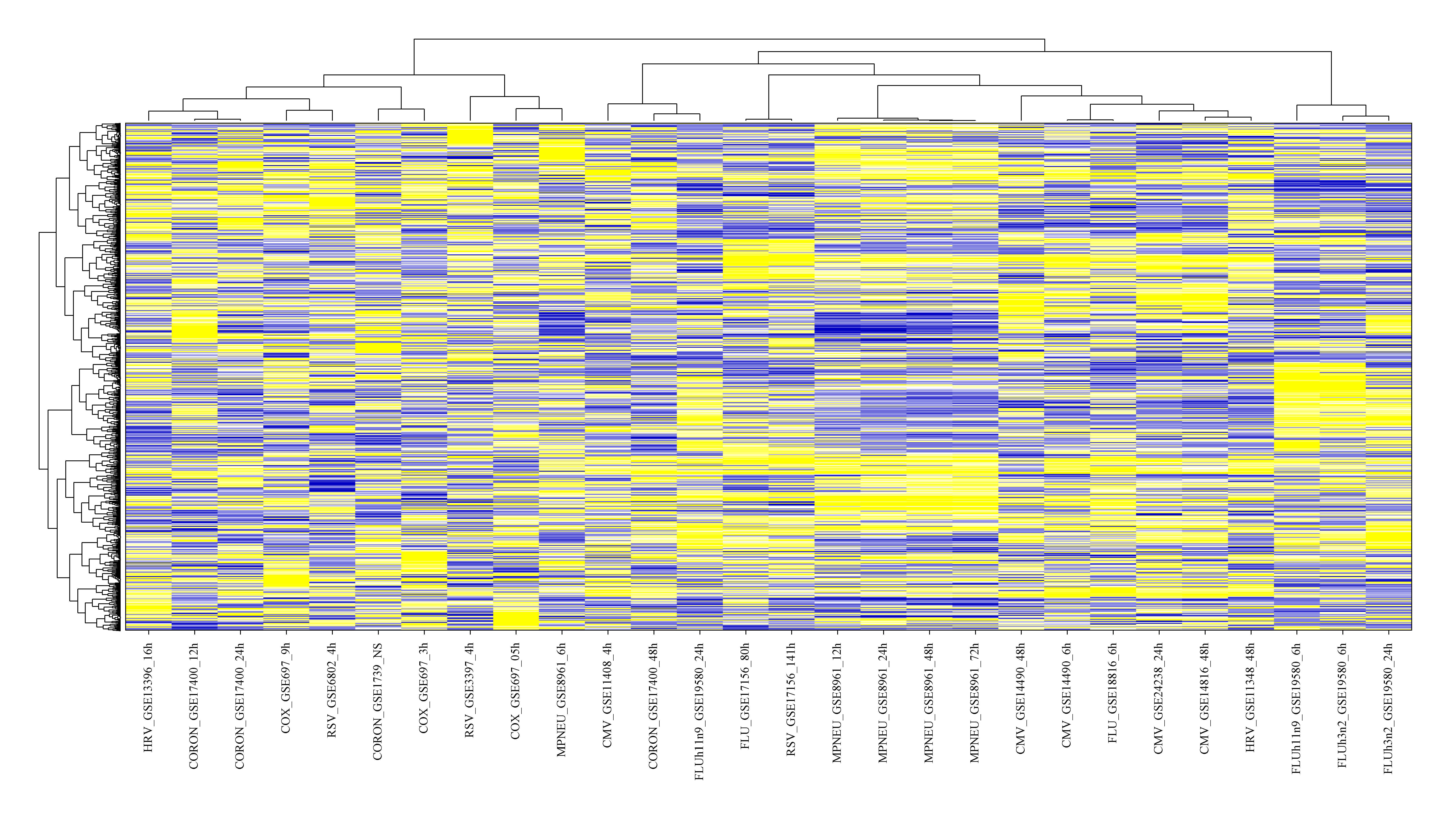

Supplement: Figure S3 — Hierarchical clustering of the fold change values on genes differentially expressed in at least 7 comparisons. The horizontal axis contains each of the 28 different comparisons labeled by virus, GSE and time point. The vertical axis shows clustering of 1,274 genes that are differentially expressed in at least 7 of the 28 comparisons and have an expression value in at least 26 comparisons. Yellow indicates a fold change value of 3.0 or greater; blue indicates a fold change value of −3.0 or less; white indicates a fold change value of 0.0. (TIF) [file pone.0033174.s003.tif]

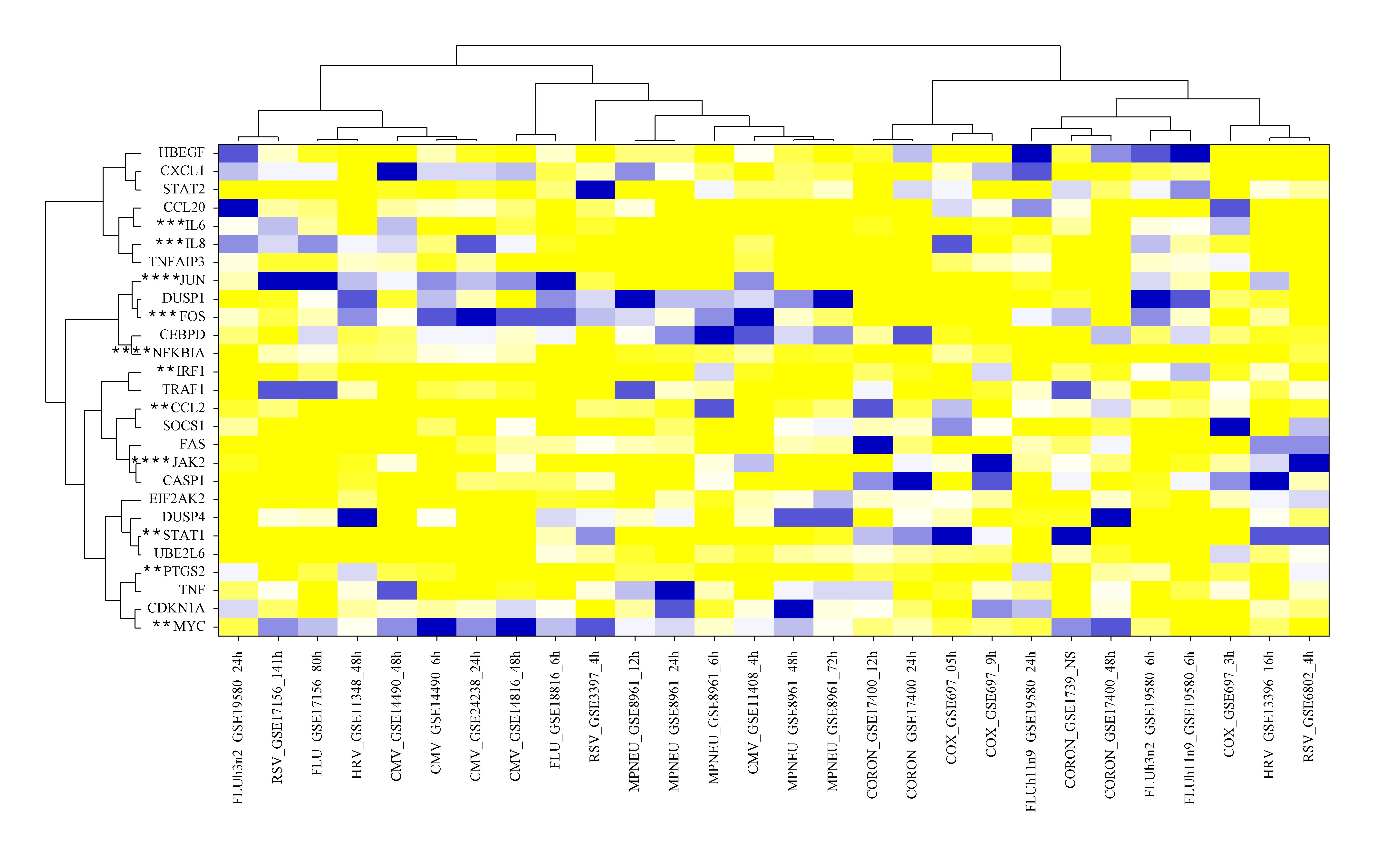

Supplement: Figure S4 — Hierarchical clustering of the fold change values on genes from top pathways with an NVE of at least 6 viruses ( Table 3 ). The horizontal axis contains each of the 28 different comparisons labeled by virus, GSE and time point. The vertical axis shows the clustering of 27 genes from the top five and Parkin-UPS pathways that have an NVE of at least 6 and have an expression value in at least 26 comparisons. For genes present in more than one of the five pathways, the number of participating pathways is indicated by the count of “*” before the gene name. Color scheme is as described for Figure S3. (TIF) [file pone.0033174.s004.tif]

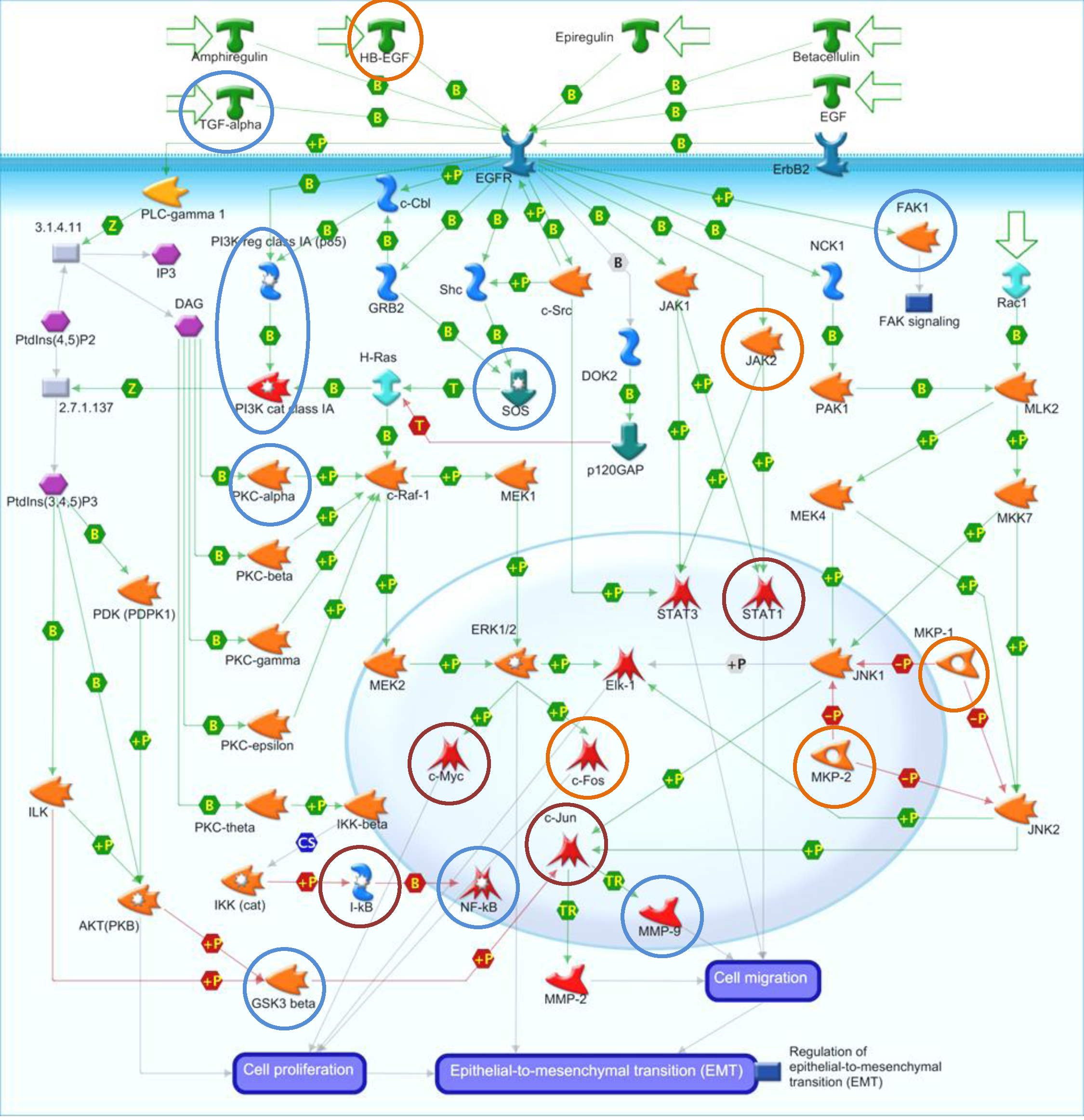

Supplement: Figure S5 — Epidermal Growth Factor Receptor signaling pathway with viral frequency. Viral frequencies superimposed for each of most frequently differentially expressed proteins, where red circles are differential expression of genes by 7 viruses, orange circles are differential expression of genes by at least 6 viruses, and blue circles are differential expression of genes by 5 viruses. See MetaCore website at http://www.genego.com/pdf/MC_legend.pdf for figure legend and Table S4 for pathway map gene products' corresponding HUGO gene names. (TIF) [file pone.0033174.s005.tif]

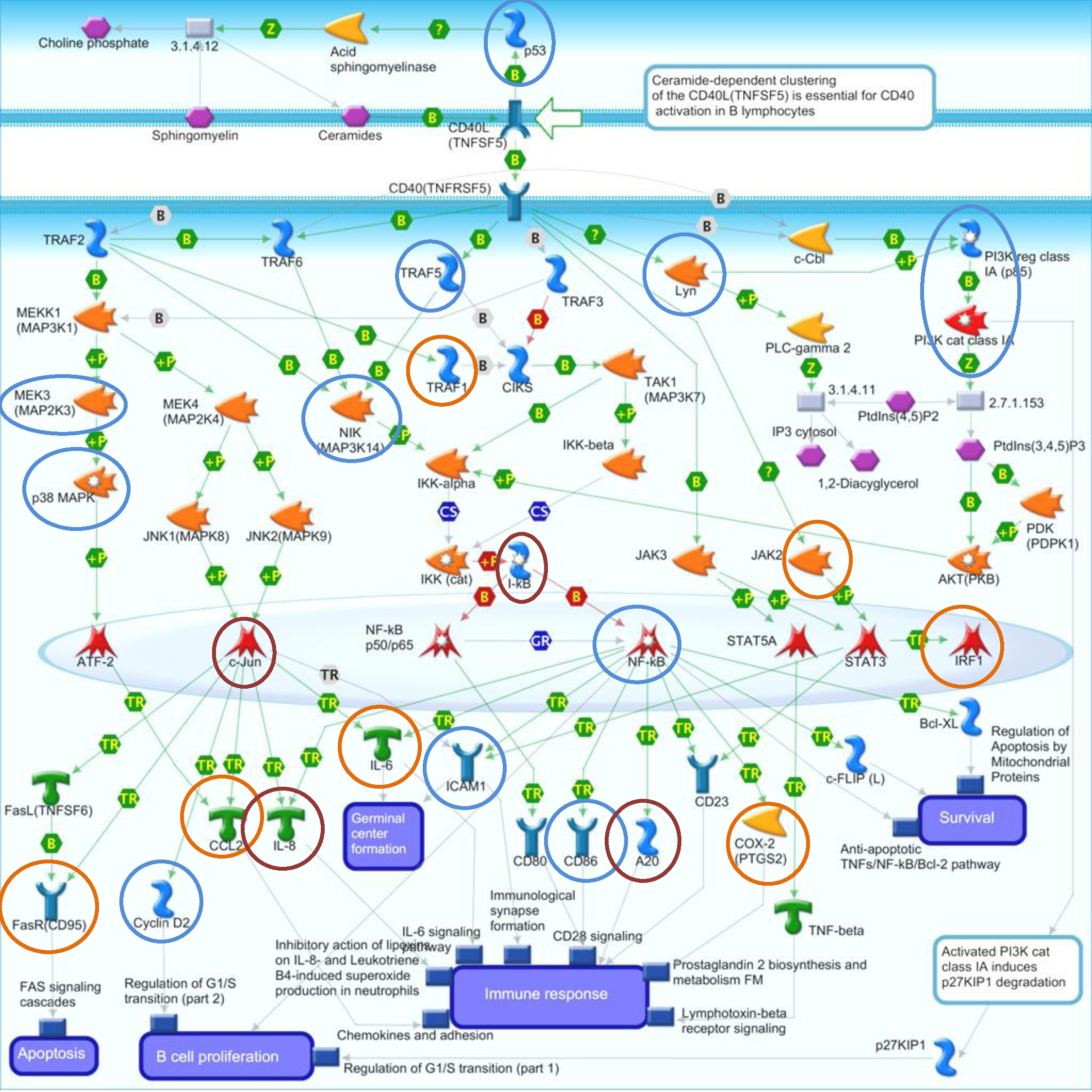

Supplement: Figure S6 — CD40 signaling pathway with viral frequency. Viral frequencies superimposed for each of most frequently differentially expressed proteins, where red circles are differential expression of genes by 7 viruses, orange circles are differential expression of genes by at least 6 viruses, and blue circles are differential expression of genes by 5 viruses. See MetaCore website at http://www.genego.com/pdf/MC_legend.pdf for figure legend and Table S4 for pathway map gene products' corresponding HUGO gene names. (TIF) [file pone.0033174.s006.tif]

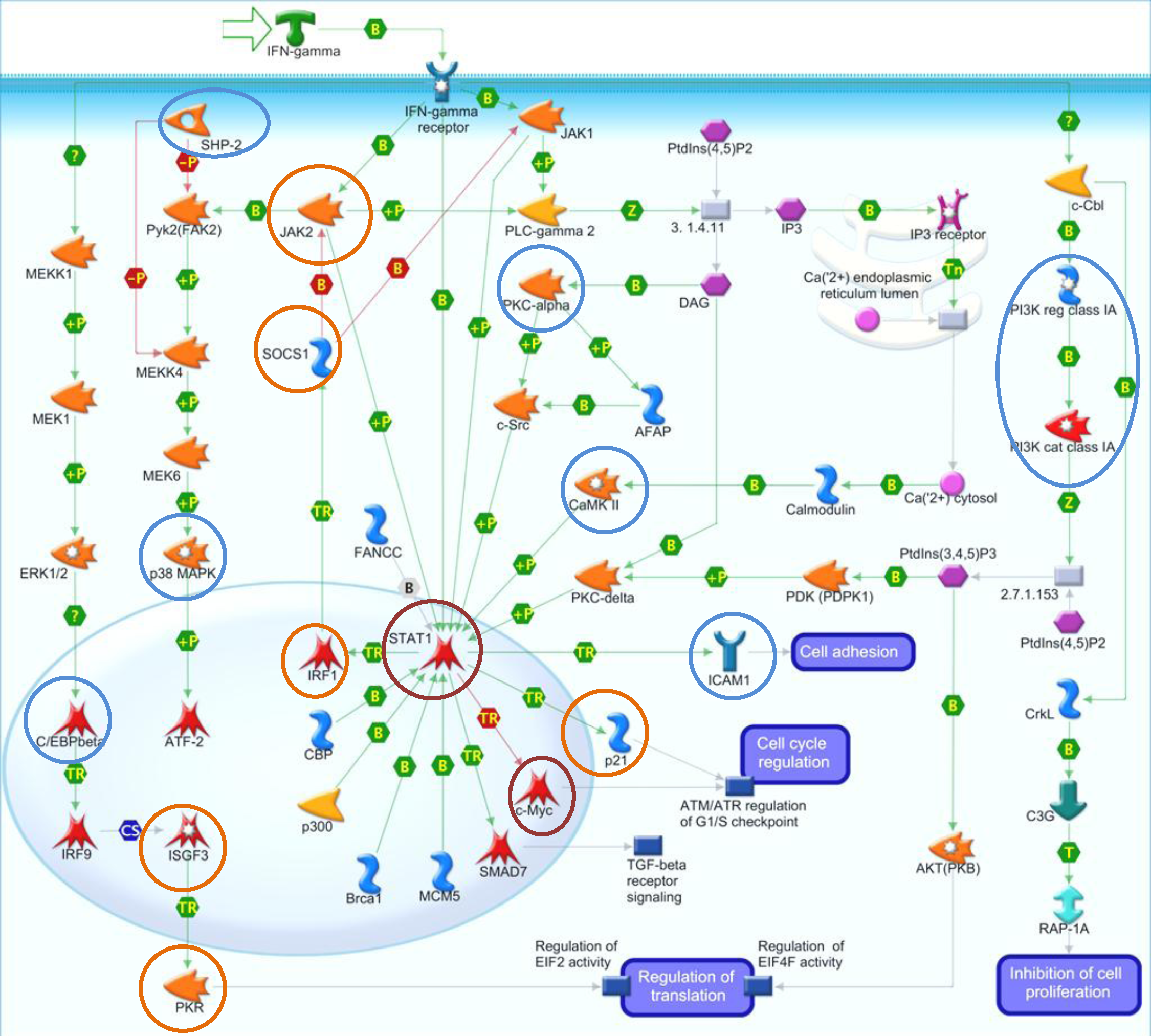

Supplement: Figure S7 — Interferon-gamma signaling pathway with viral frequency. Viral frequencies superimposed for each of most frequently differentially expressed proteins, where red circles are differential expression of genes by 7 viruses, orange circles are differential expression of genes by at least 6 viruses, and blue circles are differential expression of genes by 5 viruses. See MetaCore website at http://www.genego.com/pdf/MC_legend.pdf for figure legend and Table S4 for pathway map gene products' corresponding HUGO gene names. (TIF) [file pone.0033174.s007.tif]

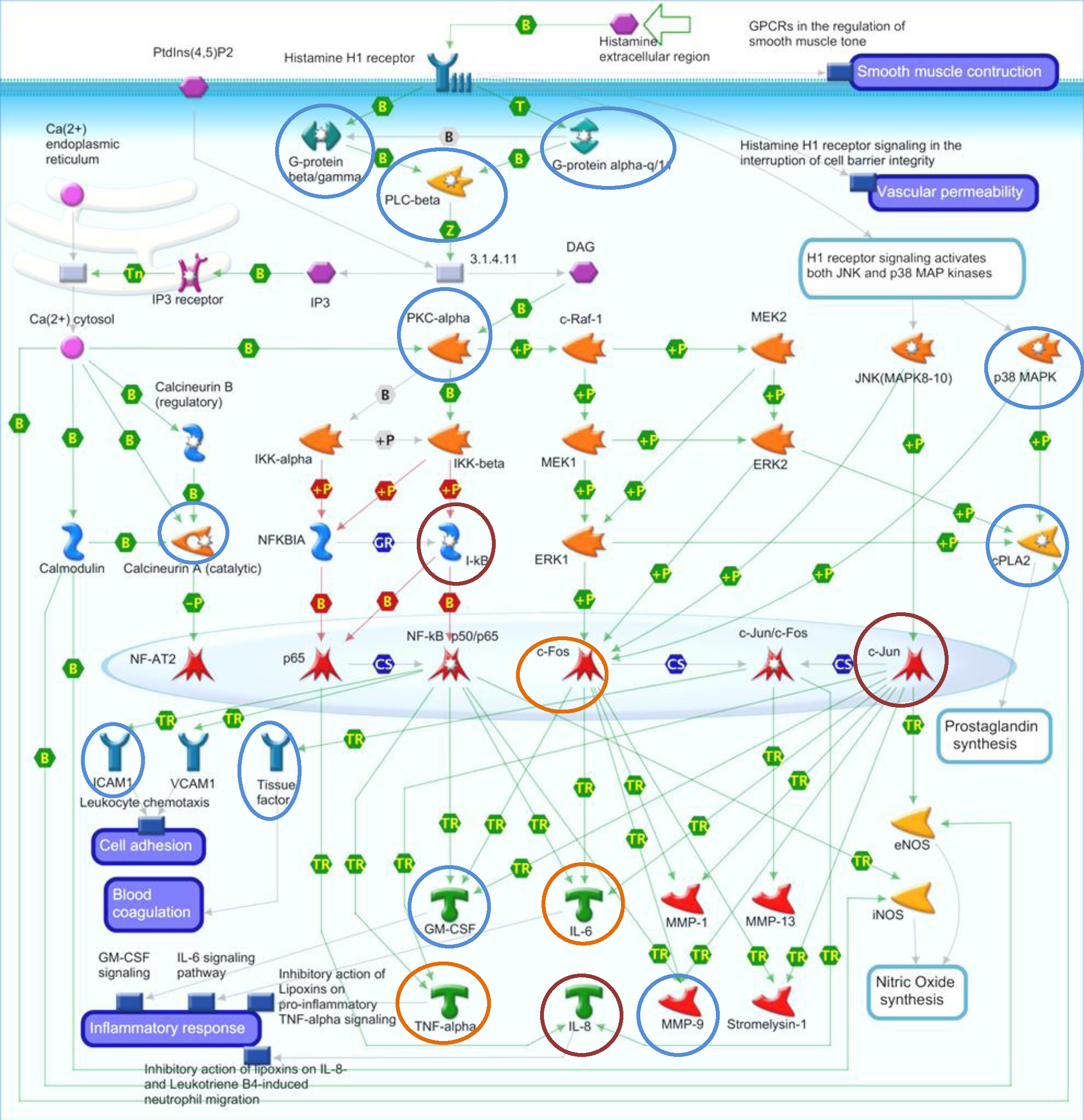

Supplement: Figure S8 — Histamine Receptor H1 signaling pathway with viral frequency. Viral frequencies superimposed for each of most frequently differentially expressed proteins, where red circles are differential expression of genes by 7 viruses, orange circles are differential expression of genes by at least 6 viruses, and blue circles are differential expression of genes by 5 viruses. See MetaCore website at http://www.genego.com/pdf/MC_legend.pdf for figure legend and Table S4 for pathway map gene products' corresponding HUGO gene names. (TIF) [file pone.0033174.s008.tif]

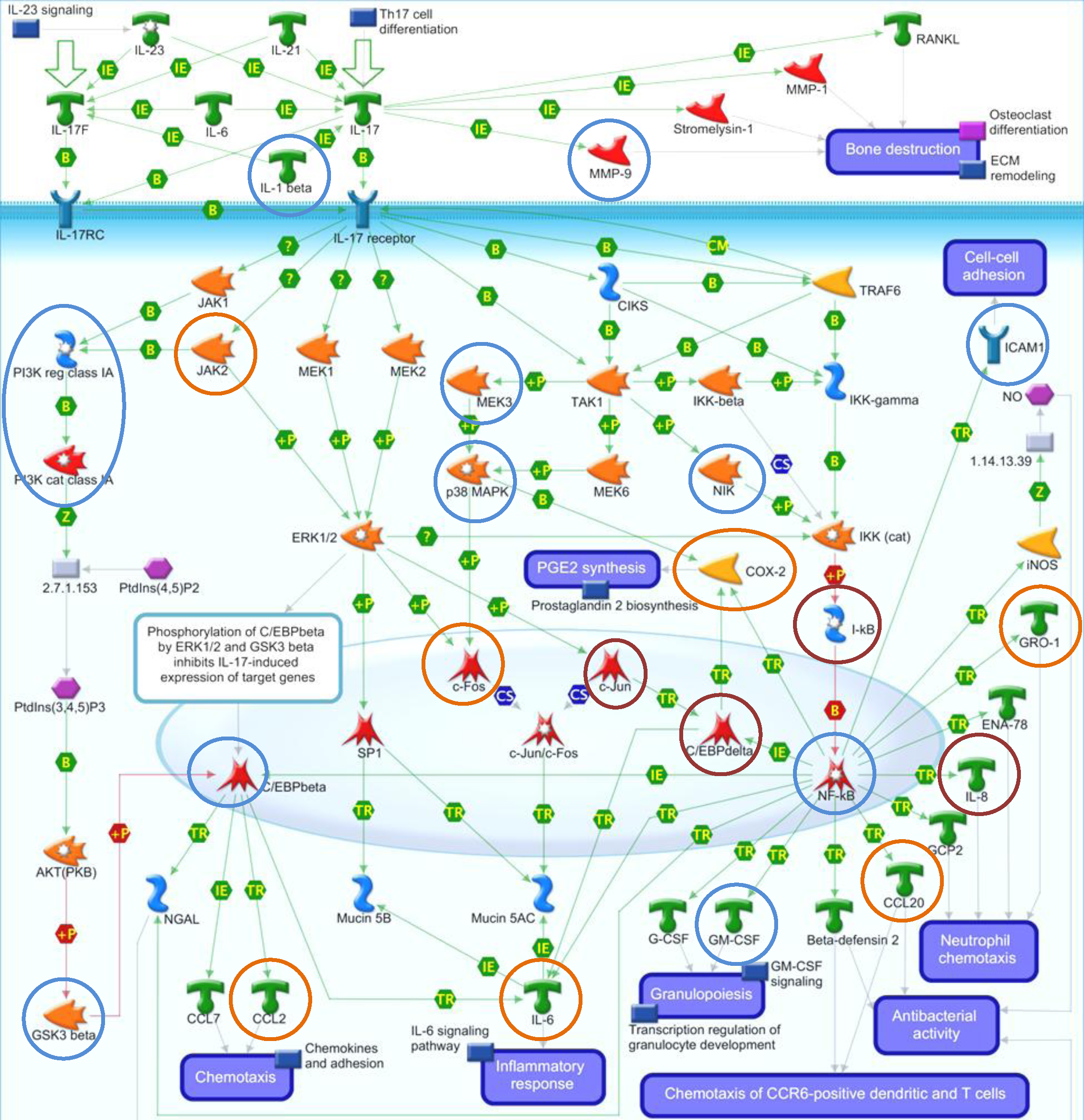

Supplement: Figure S9 — Interleukin-17 signaling pathway with viral frequency. Viral frequencies superimposed for each of most frequently differentially expressed proteins, where red circles are differential expression of genes by 7 viruses, orange circles are differential expression of genes by at least 6 viruses, and blue circles are differential expression of genes by 5 viruses. See MetaCore website at http://www.genego.com/pdf/MC_legend.pdf for figure legend and Table S4 for pathway map gene products' corresponding HUGO gene names. (TIF) [file pone.0033174.s009.tif]
